# Supplementary material for: Nonlinear optical extreme learner via data reverberation with incoherent light
Source: Sci Adv. 2026 Feb 11;12(7):eaeb4237. doi: 10.1126/sciadv.aeb4237 (PMC12893282; doi:10.1126/sciadv.aeb4237)
Supplement: Supplementary file 1 — Supplementary Text Figs. S1 to S7 Table S1 References [file sciadv.aeb4237_sm.pdf]

Science Advances
AAAS

Supplementary Materials for

Nonlinear optical extreme learner via data reverberation with incoherent light

Bofeng Liu *et al.*

Corresponding author: Xingjie Ni, xingjie@psu.edu

Sci. Adv. **12**, eaeb4237 (2026)
DOI: 10.1126/sciadv.aeb4237

This PDF file includes:

Supplementary Text
Figs. S1 to S7
Table S1
References

Supplementary Note 1: Working principle of data reverberation

In our optical cavity, a bright pixel (value "1," no external voltage) rotates the polarization, whereas a dark pixel (value "0," with external voltage) leaves the polarization essentially unchanged. In the schematics (Figure S1), the red arrow denotes Y-polarized light and the green arrow denotes X-polarized light. The shorthand "X-LC-Y" denotes, in order, the X-polarization-selective partial mirror (X-polarizer), the LC layer, and the Y-polarization-selective partial mirror (Y-polarizer).

When light enters the cavity, the Y component is transmitted by the Y-polarizer, producing the first transmitted component $T \cdot \mathbf{A}^{[1]}$. A very small portion of the parallel polarization is reflected at the interfaces – because this contribution is minor, we neglect it here for clarity. The X component is reflected by the Y-polarizer and re-enters the cavity. During propagation, diffraction causes part of this reflected light to couple into adjacent pixels, where its polarization may or may not be rotated. The X component then reaches the X-polarizer and is transmitted, giving rise to the second transmitted component $T \cdot \mathbf{A}^{[2]}$. The Y component is reflected, passes through the LC again, diffracts, and produces the third transmitted component $T \cdot \mathbf{A}^{[3]}$.

In general, most of the reflected light retraces the same pixel, contributing to **self-nonlinear** terms, while a smaller fraction, redistributed by diffraction into neighboring pixels, gives rise to **cross-nonlinear** terms. All possible cases, including different neighboring-pixel combinations and transmission orders, are illustrated in **Figure S1**. This repeated sequence of polarization conversion, transmission, reflection, and diffraction across multiple pixels is what realizes the desired nonlinear transformation.

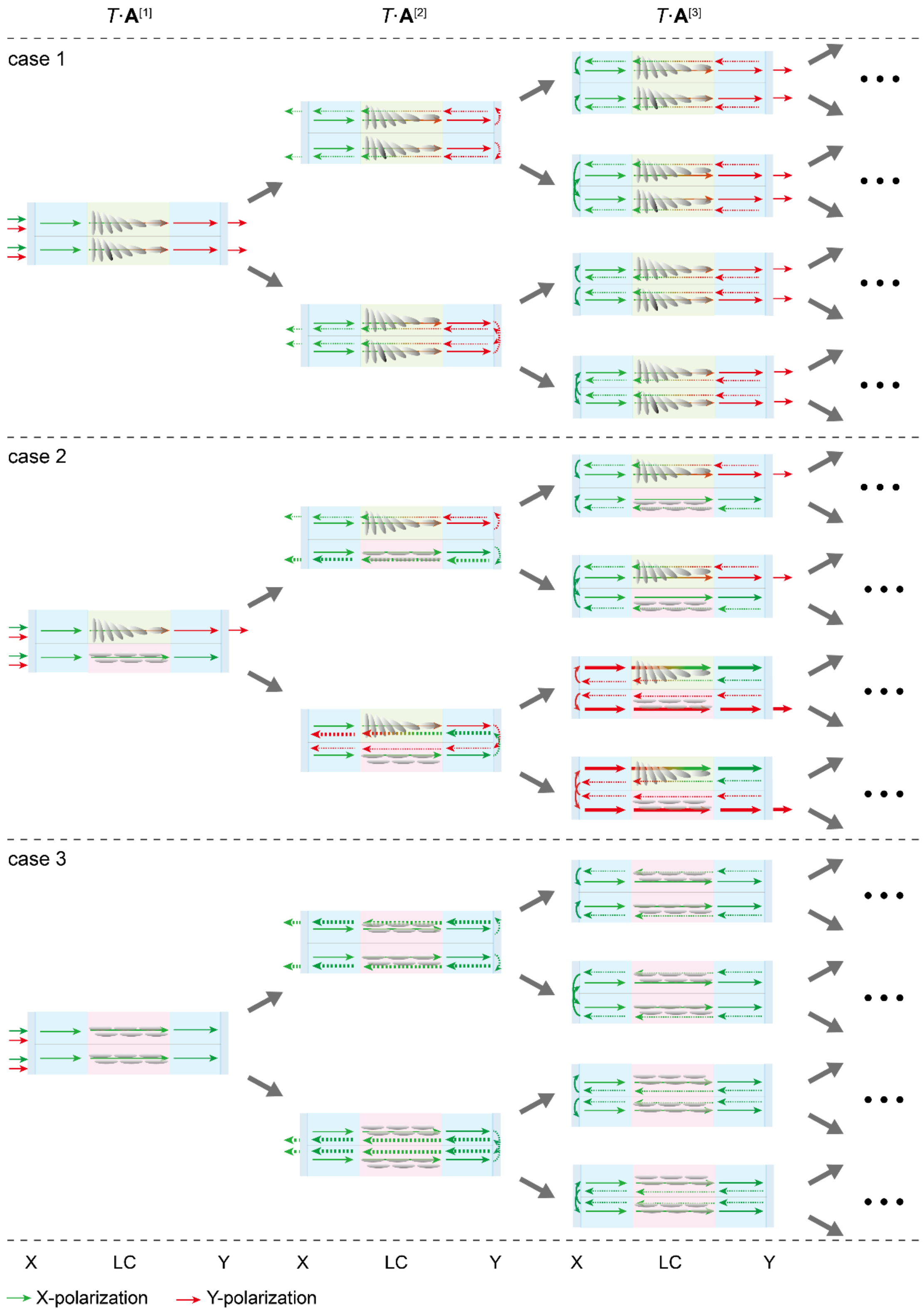

Figure.S1. First three transmitted orders arising from reflections and diffraction. The optical cavity undergoes multiple reflections and diffraction, illustrated here with two neighboring liquid-crystal (LC) pixels in three possible configurations. Red arrows denote Y-polarized light and green arrows denote X-polarized light. The symbols X, LC, and Y indicate,

respectively, the X-polarization-selective partial mirror (X-polarizer), the LC layer, and the Y-polarization-selective partial mirror (Y-polarizer). Upon reflection, most of the reflected light retraces the same pixel, contributing to self-nonlinear terms, while a smaller fraction, redistributed by diffraction into neighboring pixels, gives rise to cross-nonlinear terms. Transmission outputs are labeled as follows: $T \cdot \mathbf{A}^{[1]}$ – first (Y-polarized) transmission; $T \cdot \mathbf{A}^{[2]}$ - second (X-polarized) transmission; $T \cdot \mathbf{A}^{[3]}$ – third (Y-polarized) transmission.

Supplementary Note 2: Iterative formulation of the intensity distribution

To derive the iterative relation of the intensity distribution, let $\mathbf{A}^{[n-1]}$ denote the pixel-wise polarization-intensity profile after the $(n-1)$-th pass through the LC layer and immediately before a polarizer. We stack the per-pixel 2×2intensity matrices $\mathbf{A}_i^{[n-1]}$ for $i = 1, \ldots, N$ (with N LC pixels) as

$$\mathbf{A}^{[n-1]} = \begin{bmatrix} \mathbf{A}_1^{[n-1]} \\ \vdots \\ \mathbf{A}_i^{[n-1]} \\ \vdots \\ \mathbf{A}_N^{[n-1]} \end{bmatrix} \quad \text{(S1)}$$

So $\mathbf{A}^{[n-1]} \in \mathbb{R}^{2N\times 2}$. For the i-th LC pixel,

$$\mathbf{A}_i^{[n-1]} = \begin{bmatrix} A_{i,x}^{[n-1]} & 0 \\ 0 & A_{i,y}^{[n-1]} \end{bmatrix} \quad \text{(S2)}$$

where $A_{i,x}^{[n-1]}$and $A_{i,y}^{[n-1]}$are the intensities of the X- and Y-polarized components, respectively. This density matrix(*64*) represents incoherent light. Diagonal entries are the intensities of the orthogonal polarization states.

At the polarizer, the transmitted and reflected portions are $\mathbf{A}^{[n-1]}T$ and $\mathbf{A}^{[n-1]}R$ (with scalar transmittance T and reflectance R). The reflected field re-enters the cavity and propagates back to the LC; we model this by a convolution with the free-space Green's function $\overleftrightarrow{\mathbf{G}}$

$$\mathbf{A}'^{[n-1]} = \mathbf{A}^{[n-1]}R \circledast \overleftrightarrow{\mathbf{G}} \quad \text{(S3)}$$

Each LC pixel then modulates the polarization according to the binary pattern $\mathbf{p} \in \{0,1\}^N$: if $p_i = 0$, the polarization is unchanged; if $p_i = 1$, the X and Y components are swapped (a $90°$ polarization rotation). Let $\mathbf{P} = \mathrm{diag}(\mathbf{p})$ (size $N \times N$), I_N the $N \times N$ identity, I_2 the 2×2 identity, and $\sigma_x = \begin{bmatrix} 0 & 1 \\ 1 & 0 \end{bmatrix}$ the Pauli-X (swap) matrix. The unrotated and rotated contributions are

$$\mathbf{A}''^{[n-1]}_{\mathrm{unrot}} = [(\mathbf{I}_{\mathrm{N}} - \mathbf{P}) \otimes \mathbf{I}_2]\mathbf{A}'^{[n-1]} \tag{S4}$$

$$\mathbf{A}''^{[n-1]}_{\mathrm{rot}} = [\mathbf{P} \otimes \boldsymbol{\sigma}_x]\mathbf{A}'^{[n-1]}\boldsymbol{\sigma}_x \tag{S5}$$

where $\otimes$ indicates the Kronecker product. After this LC modulation, the field again propagates to the opposite polarizer, yielding the intensity profile after the n-th LC pass immediately before a polarizer

$$\mathbf{A}^{[n]} = \left(\mathbf{A}''^{[n-1]}_{\mathrm{rot}} + \mathbf{A}''^{[n-1]}_{\mathrm{unrot}}\right) \circledast \overleftrightarrow{\mathbf{G}} \tag{S6}$$

Combining the steps above gives the complete iterative relation

$$\mathbf{A}^{[n]} = \left\{[\mathbf{P} \otimes \boldsymbol{\sigma}_x]\left(\mathbf{A}^{[n-1]}R \circledast \overleftrightarrow{\mathbf{G}}\right)\boldsymbol{\sigma}_x + [(\mathbf{I} - \mathbf{P}) \otimes \mathbf{I}_2]\left(\mathbf{A}^{[n-1]}R \circledast \overleftrightarrow{\mathbf{G}}\right)\right\} \circledast \overleftrightarrow{\mathbf{G}} \tag{S7}$$

Supplementary Note 3: Nonlinear order analysis

The nonlinearity relation between the input patterns implemented on LCD panel and the optical output pattern is characterized by Equation (2) in main text. To analyze this nonlinearity, we employ *N* binary pixels to generate a total of 2^N possible binary input configurations, denoted as $\boldsymbol{p}^{(r)} = \left(p_1^{(r)}, p_2^{(r)}, p_3^{(r)}, \dots, p_N^{(r)}\right)$, ($r$=1,2,…,$2^N$ is the *r*-th configuration). For each input, the corresponding optical intensity pattern captured by the camera consists of *M* pixels, denoted as $\boldsymbol{A}^{(r)} = \left(A_1^{(r)}, A_2^{(r)}, A_3^{(r)}, \dots, A_M^{(r)}\right)$. The nonlinear mapping between $\boldsymbol{p}^{(r)}$ and $\boldsymbol{A}^{(r)}$ can be systematically expressed using Boolean function decomposition method(*17, 51*), leading to the following functional expansion:

$$A_m^{(r)} = f\left(p_1^{(r)}, p_2^{(r)}, p_3^{(r)}, \dots, p_N^{(r)}\right)$$
$$= c_{m;0}^{(r)} + \underbrace{\sum_{n_1=1}^{N} c_{m;n_1}^{(r)} p_{n_1}^{(r)}}_{d=1} + \underbrace{\sum_{n_1=1}^{N} \sum_{n_2=1}^{n_1} c_{m;n_1,n_2}^{(r)} p_{n_1}^{(r)} p_{n_2}^{(r)}}_{d=2}$$
$$+ \cdots + \underbrace{c_{m;1,2,\dots,N}^{(r)} p_1^{(r)} p_2^{(r)} \dots p_N^{(r)}}_{d=N} \qquad \text{(S8)}$$

Where $p_n^{(r)} \in \{-1,1\}$, m=1,2,…,M, $c_{m;n}^{(r)}$ are the expansion coefficients corresponding to each order. On the right side of Equation (S8), the different order d=1,2,…,N, of the output terms is related to the cross talk in $p^{(r)}$. The term with d=1 correspond to linear contributions, whereas high-order term with $d \geq 2$ capture nonlinear interactions (i.e. crosstalk) among the input pixels. To determine the expansion coefficients, we systematically measure the output intensity patterns corresponding to all 2^N binary input configurations and fit the model in Equation (S8) to the experimental data. Averaging $\left|c_{m;n_1,n_2,\dots,n_d}^{(r)}\right|$ over m and r for a fixed order d yields the mean expansion coefficient $\bar{c}(d)$ (Figure S2(b)).

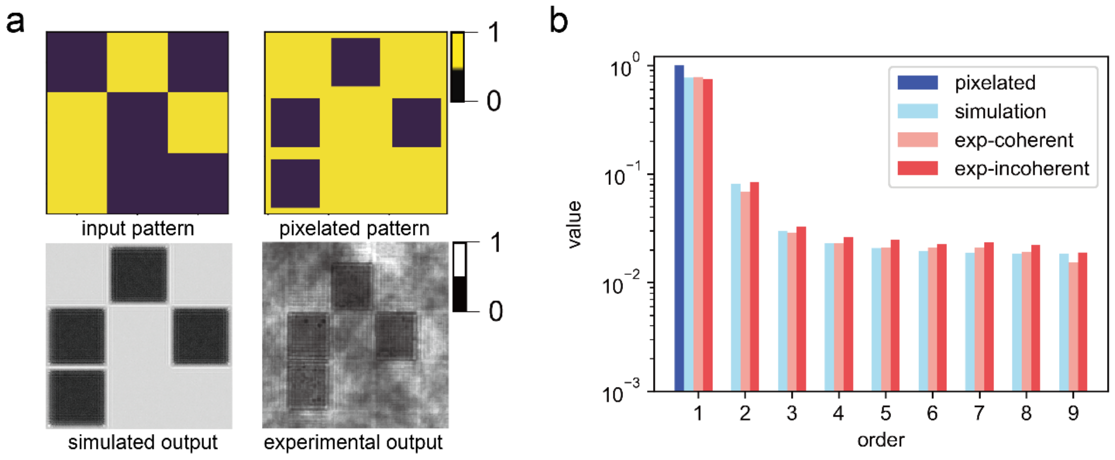

Figure S2. Analysis of nonlinear orders. (a) Boolean function analysis pattern. The input pattern is one of the 2^9 possible binary combinations produced by a 3×3 pixel array. A pixelated version mimics the LCD grid. The simulated output shows the predicted nonlinear mapping. The experimental output is recorded directly from the setup. (b) Distribution of expansion orders. The horizontal axis indicates the interaction order d, and the vertical axis shows the magnitude of the corresponding expansion coefficients. First-order (linear) coefficients dominate, followed by high-order (nonlinear) terms.

Figure S2 presents the Boolean function analysis procedure used to quantify the input-output mapping's nonlinearity. The input pattern contains N=3×3 pixels, yielding 2^9 possible distinct binary configurations. Pixelated inputs, simulated outputs, and experimentally obtained images share the same physical extent. The first two have M = 1500 × 1500 pixels, while the experimental images have M = 200 × 200 pixels. Figure S2(b) shows the expansion coefficients of Equation (S8). Mapping the original pattern onto the LCD grid is a purely linear (matrix-multiplication) step, so its energy appears only in the first-order term. In contrast, both simulation and experiment exhibit substantial higher-order contributions, revealing strong nonlinear interactions in the optical cavity.

Supplementary Note 4: Neural network design

Conventional multilayer neural networks contain at least one hidden layer that provides a nonlinear mapping (Figure S3(a)). The necessity of this nonlinearity follows from the universal approximation theorem (*36-38*), which can be expressed as,

$$\forall \epsilon > 0, \forall f(x): K \Rightarrow \mathbb{R}, f \in C^0, \exists g(x) = \sum_{j=1}^{M} \left(a_j \sigma\left(w_j \cdot x + b_j\right)\right)$$

$$\text{s.t. } \forall x \in K, |f(x) - g(x)| < \epsilon \qquad \text{(S9)}$$

Where $K \subset \mathbb{R}$ is a compact input domain, M is the number of hidden neurons, a_j are the weight of the output layer, w_j are the weight of the hidden layer, b_j are biases, and $\sigma(\cdot)$ is a nonlinear activation function. Building on this principle, we design an optical neural network that introduces *data-reverberation nonlinearity* to compensate for the intrinsically linear response of low-power optical hardware. First, the input image is linearly encoded, emulating the network's initial linear layer. This encoded pattern is displayed on a binary LCD panel that modulates an incident beam. Repeated reflections within the LCD cavity – each time the light passes through the encoded pattern – generate the required nonlinearity. A camera then captures the resulting nonlinearly mapped intensity distribution at the plane of the LCD. Finally, the recorded data are fed to a last linear layer to perform image classification and XOR computation. The overall concept is illustrated in Figure S3(b).

To ensure a fair comparison with the proposed nonlinear optical network, we implemented a digital linear baseline with one hidden layer and no nonlinear activation functions. For MNIST, the network architecture includes 400 input neurons, 300 hidden neurons, and 10 output neurons, corresponding to 300×400 + 10×300 + 10 = 123,010 trainable parameters. For the CMNIST and EMNIST datasets, we use the same 400-300 trunk with output sizes of 15 and 26, resulting in 124,515 and 127,826 parameters, respectively. Details of the model training are provided in the *Materials and Methods* section of the main text.

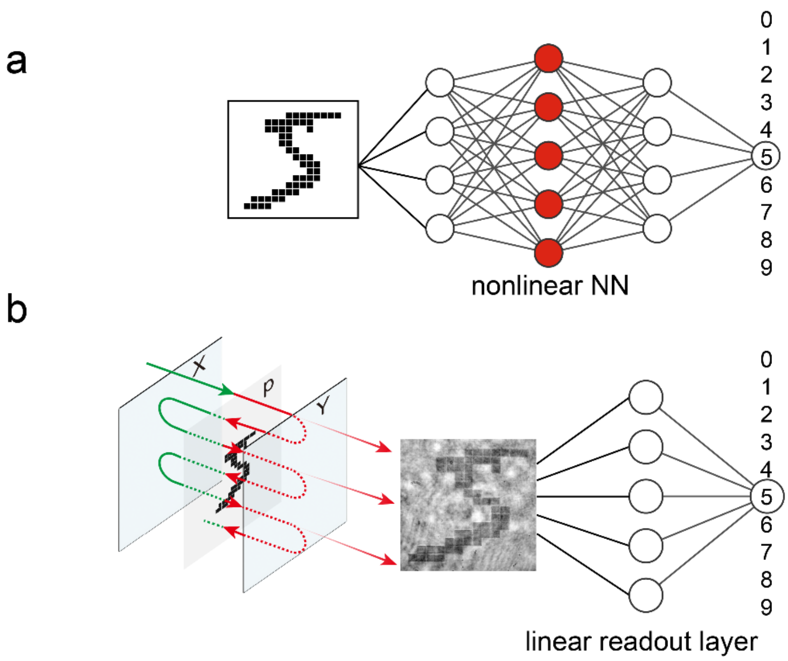

Figure S3. Comparison between digital neural networks and the optical extreme learner. (a) A standard neural network includes at least three stages: a first linear layer, a nonlinear activation layer (highlighted in red), and a final linear layer. (b) In the proposed architecture, the nonlinear activation is provided optically: light passes through the LCD cavity, whose nonlinear mapping through data reverberation replaces the explicit activation layer.

Figure S4 shows the training-loss curves for the MNIST, Chinese MNIST, and EMNIST datasets. In every case, the features produced by the optical nonlinear mapping follow a lower loss trajectory – comparable to that of a fully nonlinear digital network – during image classification.

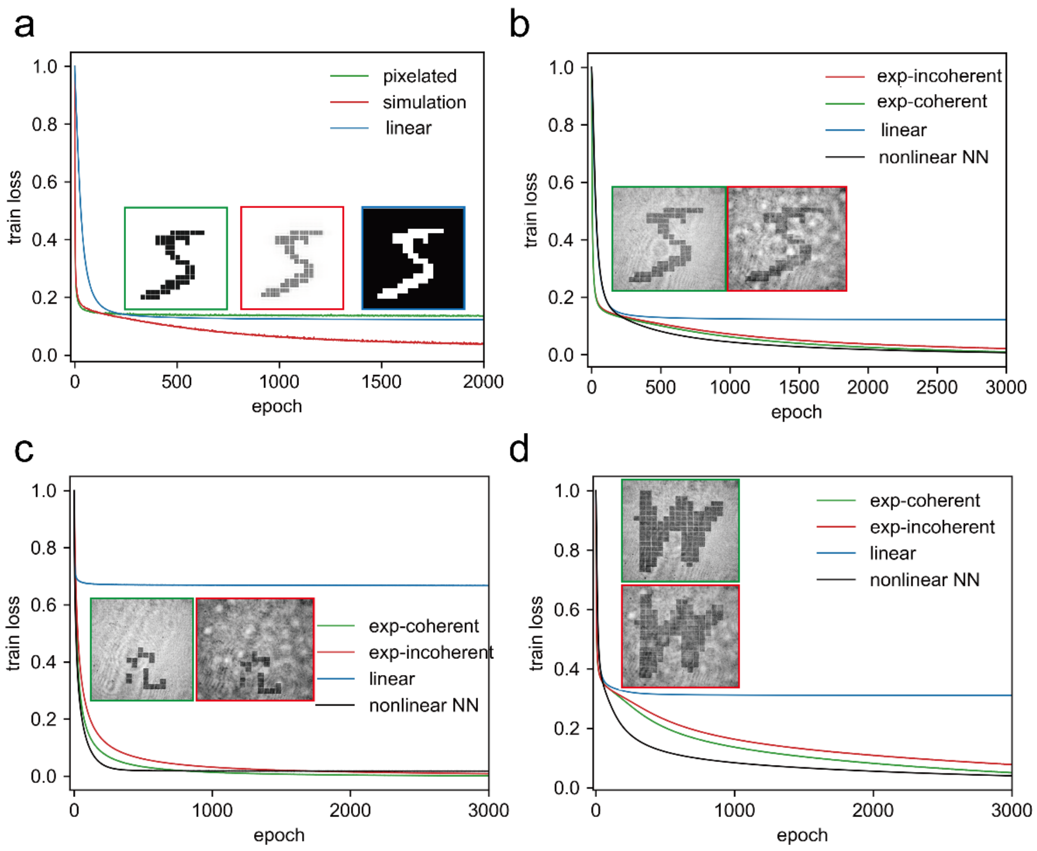

Figure S4. Training-loss curves for image classification. (a) MNIST simulation results: linear network on the original dataset (blue), linear network on the pixelated dataset (green), and linear network on the simulation output (red). (b) MNIST experimental results: optical extreme learner with laser illumination (green) and with white-light illumination (red), compared with linear (blue) and nonlinear (black) digital networks on the original dataset. (c) Chinese MNIST (CMNIST) experimental results: same color scheme and comparisons as in (b). (d) EMNIST experimental results: same color scheme and comparisons as in (b).

Supplementary Note 5: 10-fold cross-validation

Because the Chinese MNIST dataset lacks predefined training and test splits, we adopted 10-fold cross-validation to obtain a robust and unbiased estimate of model performance. In the 10-fold cross-validation protocol, the dataset is randomly divided into ten equal folds. In each iteration, one fold serves as the validation set while the remaining nine folds form the training set. This procedure is repeated ten times, so every sample is used exactly once for validation. Accuracy curves from all folds are then averaged to yield the final evaluation. This strategy reduces evaluation bias and variance and maximizes use of limited data – particularly valuable for non-standard datasets that lack predefined train-test splits (*52, 53*).

We applied this protocol to the Chinese MNIST dataset. The resulting accuracy and loss curves, shown in Figure S5, exhibit high consistency across folds, indicating stable training behavior and reliable generalization. These results demonstrate that the optical extreme learner is insensitive to a specific choice of training-validation split, supporting the validity of our evaluation methodology.

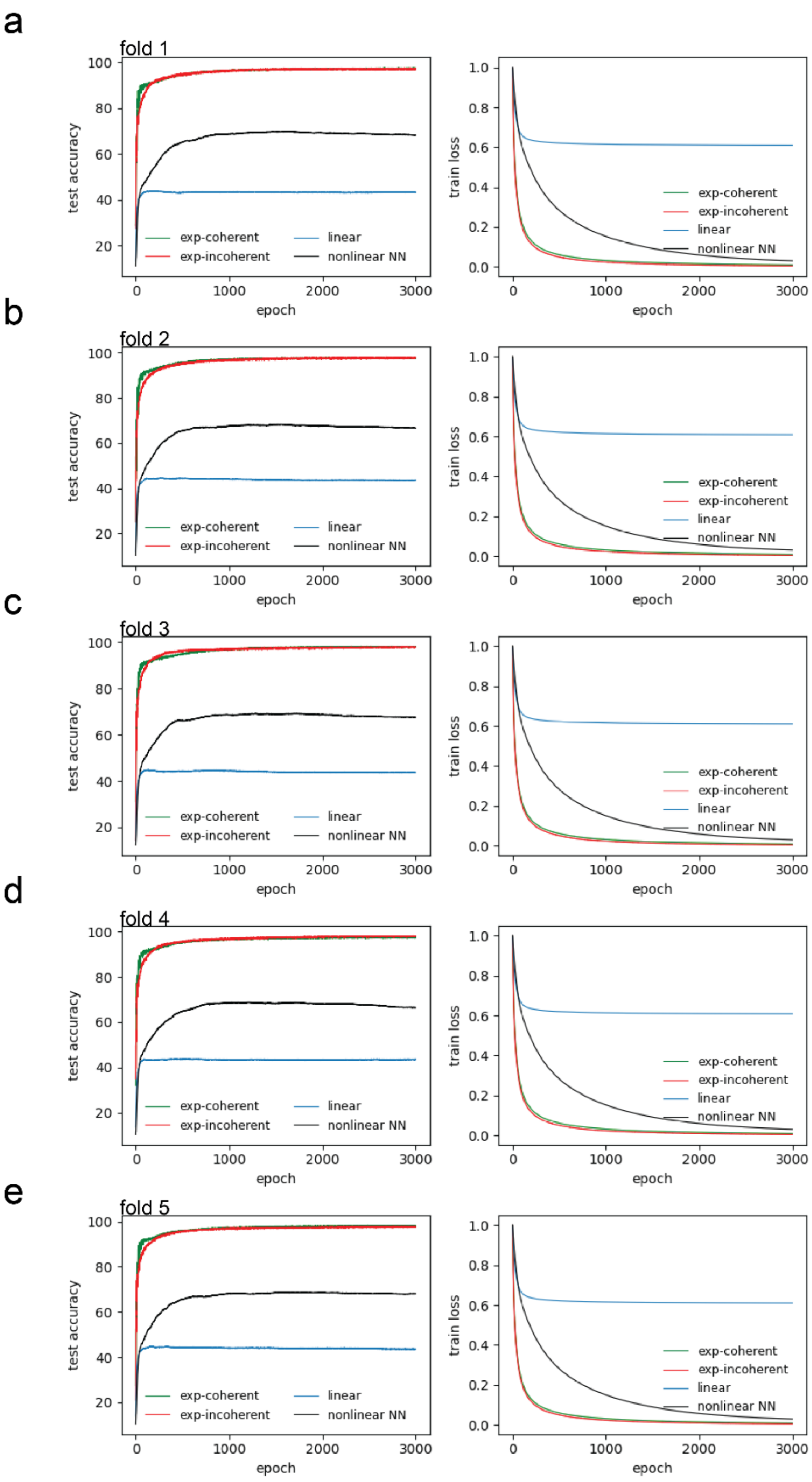

a
fold 1
test accuracy
train loss
epoch
exp-coherent
exp-incoherent
linear
nonlinear NN
b
fold 2
test accuracy
train loss
epoch
exp-coherent
exp-incoherent
linear
nonlinear NN
c
fold 3
test accuracy
train loss
epoch
exp-coherent
exp-incoherent
linear
nonlinear NN
d
fold 4
test accuracy
train loss
epoch
exp-coherent
exp-incoherent
linear
nonlinear NN
e
fold 5
test accuracy
train loss
epoch
exp-coherent
exp-incoherent
linear
nonlinear NN

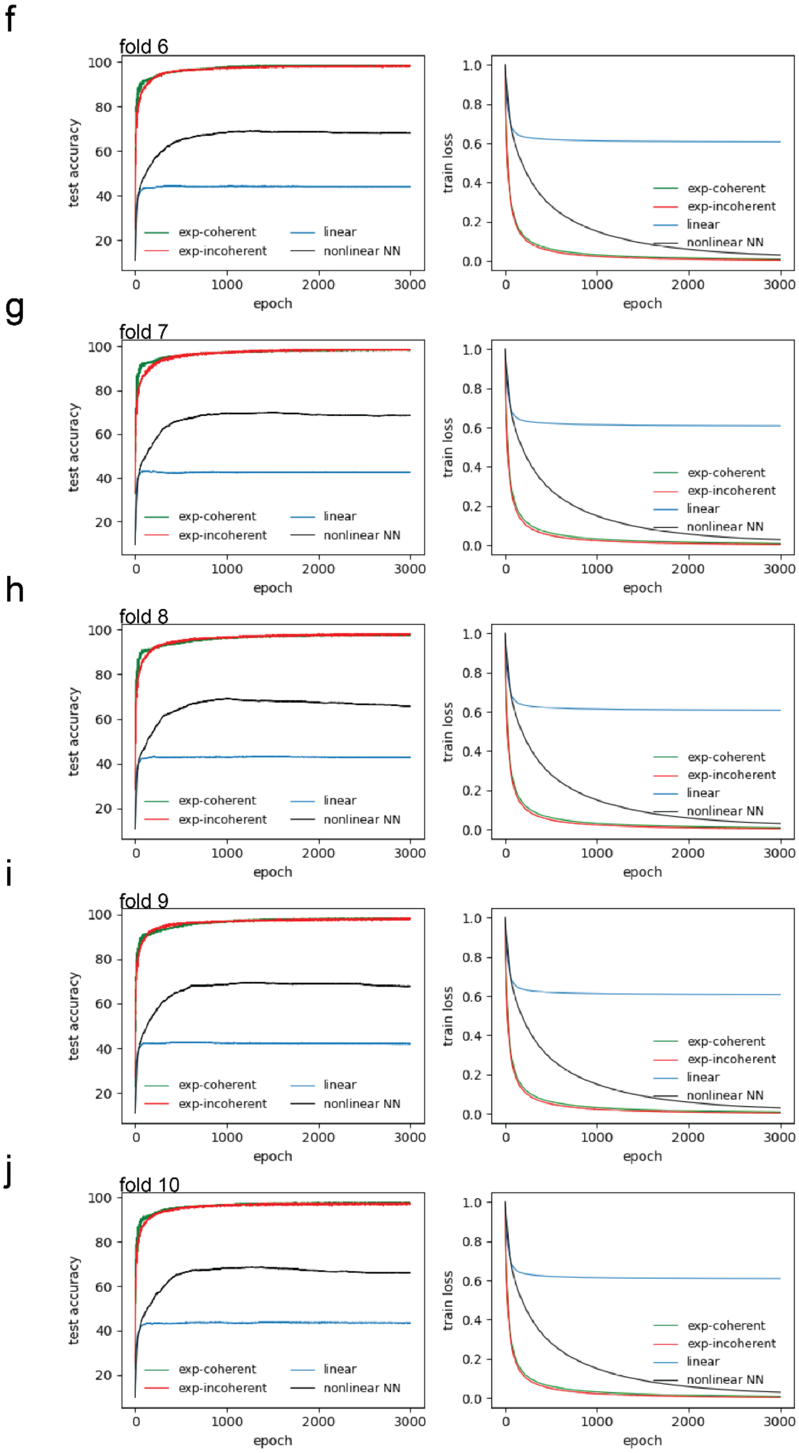

Figure S5. 10-fold cross-validation on Chinese MNIST dataset. (a)-(j) The test-accuracy and loss curves for each of the ten individual folds.

Supplementary Note 6: XOR operation test

XOR is the archetypal linearly inseparable problem introduced by Minsky and Papert (*55*). As shown in Figure S6(a), the outputs of AND, NAND, and OR gates can be separated by a single hyperplane, whereas XOR outputs cannot. To evaluate our system's nonlinearity, we split each 20 × 20 input image into the two 19 × 19 regions outlined in green and red in Figure 4(a) of the main text. We then form a new 19 × 19 target image whose pixels are the XOR of diagonally opposite pixels from the two sub-images (Figure S6(b)). Because this transformation is nonlinear, a purely linear network cannot predict the target pixels correctly, while our optical extreme leaner – whose optical cavity supplies the nonlinearity – can perform the task accurately. Figure S6(c) compares the training and test losses for the various configurations, and representative outputs are displayed.

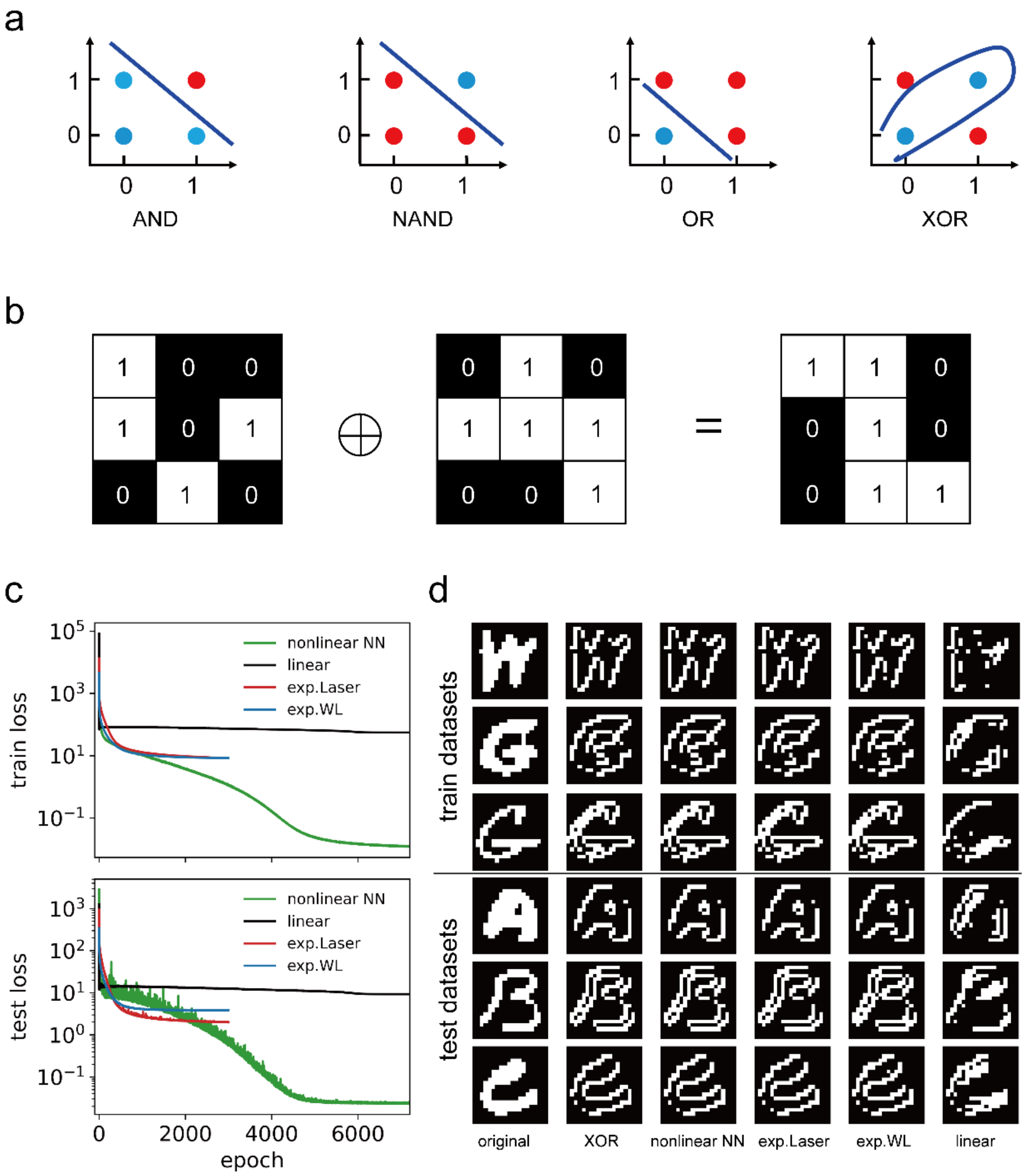

Figure S6. XOR train and test. (a) Truth tables for AND, NAND, OR, and XOR. The first three are linearly separable, whereas XOR is linearly inseparable. (b) Construction of the 19×19 target image by pixel-wise XOR between diagonally opposite pixels of the two sub-images. (c) Training and test loss curves: nonlinear digital network on the original dataset (green), linear digital network (black), optical nonlinear mapping under laser illumination (red), and optical nonlinear mapping under white-light illumination (blue). (d) Example training and test outputs for each configuration.

Supplementary Note 7: Performance comparison with existing optical nonlinear systems

To place our work in the context of prior research, we provide a direct performance comparison between our approach and representative optical nonlinear systems reported in the literature, summarized in Table S1. Different mechanisms have been employed to introduce nonlinearity, including data repeating(*48*), optical-to-electrical conversion (*26, 65*), Kerr effect (*66*), and reverse saturable absorption (*67*). The classification accuracies on the MNIST dataset achieved by these methods range from 88% to 97%. Our proposed data reverberation approach achieves a classification accuracy of 97%, which is on par with or surpasses the best results from prior optical nonlinear implementations.

This comparison highlights that the nonlinearity based on data reverberation not only provides competitive performance but also avoids the drawbacks associated with conventional nonlinear mechanisms such as high optical power requirements (Kerr effect), material constraints (reverse saturable absorption), or reliance on electro-optical conversions. Therefore, the proposed method offers a promising and energy-efficient pathway toward realizing nonlinear functionality in large-scale optical neural networks.

Table. S1. The direct performance comparison with other types of optical nonlinear systems.

	Source of Nonlinearity	MNist
Ref(*48*)	Data repeating	88%
Ref(*26*)	Optical-to-electrical conversion	92%
Ref(*65*)	Optical-to-electrical conversion	94%
Ref(*66*)	Kerr effect	95%
Ref(*67*)	Reverse Saturable Absorption	97%
This work	Data reverberation	97%

Supplementary Note 8: Energy consumption and the optical readout layer design

1. Energy consumption estimation of linear networks

To estimate the upper-bound energy consumption of the digital readout layer, we consider a realistic embedded deployment. As a representative edge platform, we use the NVIDIA Jetson AGX Orin, which is widely adopted in embedded AI and has well-documented energy and computational characteristics.

The Jetson AGX Orin delivers up to 200 TOPS (INT8 operations) with a maximum power consumption of 40W. For a readout layer containing approximately 10^5 trainable parameters, one multiply-accumulate (MAC) operation is needed for each parameter. Since 1 MAC $\approx$ 2 INT8 operations, the per-inference workload is $\approx 2\times10^5$ INT8 operations. The estimated time for each inference is

$$t \approx \frac{2\times10^5 \text{ops}}{200\times10^{12}\text{ops}} = 1\times10^{-9}\text{s}$$

at P = 40W, and the corresponding energy cost per inference is

$$\mathcal{E} = Pt \approx 40\,\text{W}\times1\,\text{ns} = 40\,\text{nJ}$$

This is a conservative upper bound. Practical inference seldom sustains peak TOPS at peak power, and power-scaling, memory effects, and workload distribution typically reduce the true energy well below this value.

It is worth emphasizing that this estimation approach follows standard benchmarking practices widely adopted in evaluating the energy efficiency of digital inference platforms. By taking the worst-case assumption (maximum power × theoretical peak runtime), our analysis ensures that the reported value is an upper limit. Even under this deliberately pessimistic assumption, the digital readout's cost remains in the nanojoule range – negligible compared with typical millijoule-level budgets reported for digital nonlinear networks(*68-70*). Thus, the digital readout is not an energy bottleneck.

Furthermore, because the readout is purely linear, it can also be realized all-optically, e.g., with metasurfaces(*40, 57, 58, 71*),providing functional equivalence to the digital

layer while operating at the speed of light and, in principle, incurring vanishing incremental electronic energy (passive propagation).

2. All-optical readout layer: design and simulation

To demonstrate feasibility, we simulate a metasurface-based readout that takes in the incoherent-intensity output of the optical cavity and maps it to class labels by free-space propagation, eliminating the residual digital computation and energy consumption associated with digital readout. A cascade of five diffractive layers (an optical diffractive neural network, ODNN (*57*)) is trained so that the transmitted field constructively focuses onto designated spots on the detection plane – one Gaussian target per class (digits 0-9) (Figure S7(a)). The metasurfaces then engineered to implement the learned phase profiles, and classification is obtained directly from the spatial energy distribution (winner-take-all across detector spots), without digital post-processing.

During training, each cavity output pattern is represented with amplitude plus a random phase to form a complex field; to suppress coherence artifacts, we average over 20 independent random phases per sample. Free-space propagation between layers is modeled with the band-limited angular spectrum method (*72*). Phase profiles of the diffractive layers are optimized by gradient-based backpropagation to maximize energy concentration in the correct detector zone. The resulting metasurface phases and simulated readout patterns are shown in Figure S7(b). On MNIST, the all-optical readout attains 96% accuracy, comparable to the digital linear readout.

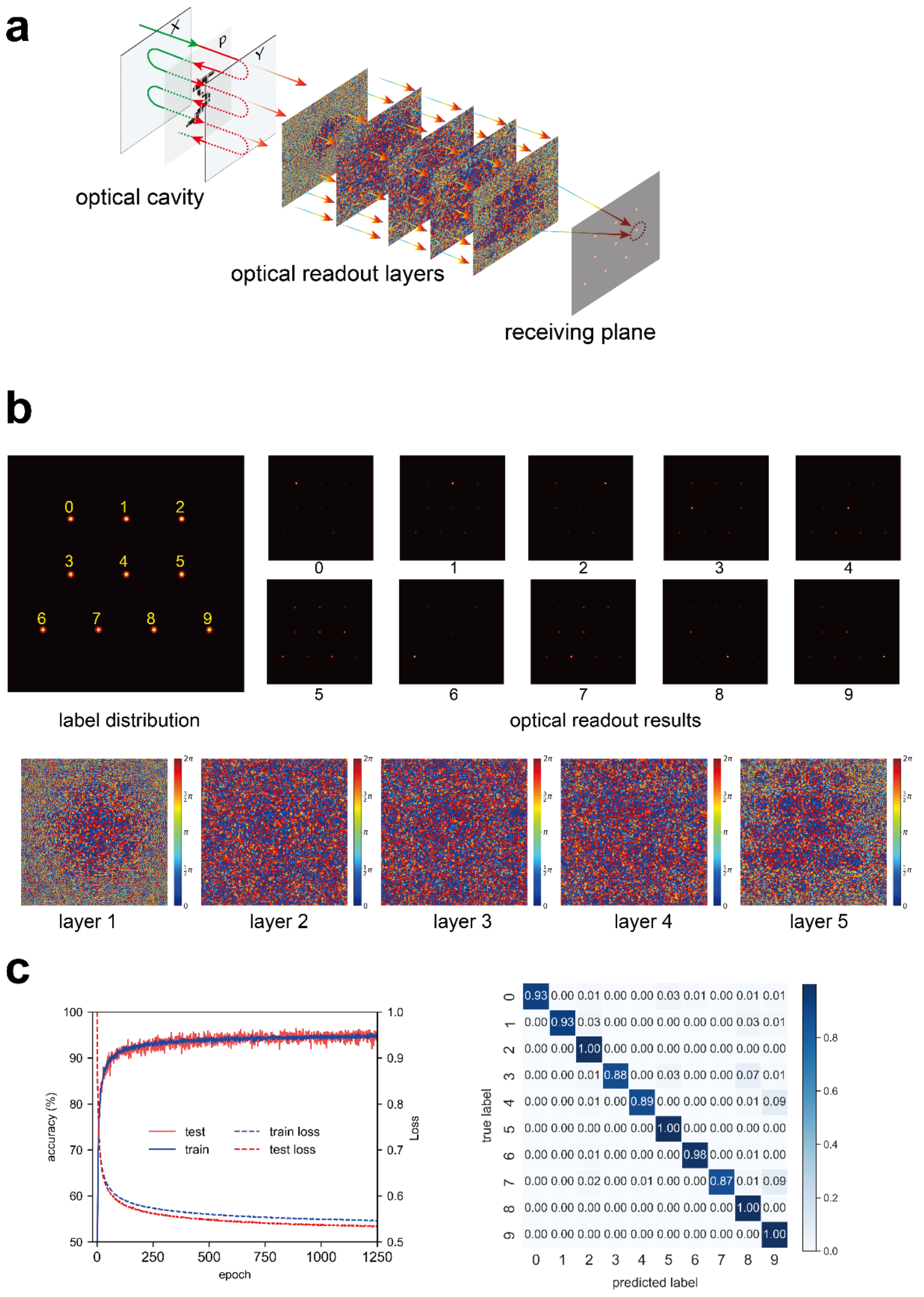

Figure S7. All-optical readout layer. (a) Schematic of the end-to-end system, consisting of the optical cavity (main text) followed by the metasurface readout layer. (b) Training labels, simulated optical readout results, and optimized phase profiles of the readout metasurfaces. (c) MNIST performance: classification accuracy and confusion matrix obtained with the optical readout.

REFERENCES

1. E. Abbe, Beiträge zur Theorie des Mikroskops und der mikroskopischen Wahrnehmung. *Archiv für mikroskopische Anatomie* **9**, 413–468 (1873). [Contributions to the Theory of the Microscope and the Nature of Microscopic Vision].

2. P. Ambs, Optical computing: A 60-year adventure. *Adv. Opt. Technol.* **2010**, 372652 (2010).

3. P. M. Duffieux, *The Fourier Transform and Its Applications to Optics* (John Wiley & Sons,1983).

4. J. W. Goodman, A. R. Dias, L. M. Woody, Fully parallel, high-speed incoherent optical method for performing discrete Fourier transforms. *Opt. Lett.* **2**, 1–3 (1978).

5. D. Psaltis, D. Brady, X.-G. Gu, S. Lin, Holography in artificial neural networks. *Nature* **343**, 325–330 (1990).

6. S. L. Yeh, R. C. Lo, C. Y. Shi, Optical implementation of the Hopfield neural network with matrix gratings. *Appl. Optics* **43**, 858–865 (2004).

7. P. E. Ceruzzi, *A History of Modern Computing* (MIT press, 2003).

8. D. R. Solli, B. Jalali, Analog optical computing. *Nat. Photon.* **9**, 704–706 (2015).

9. J. Feldmann, N. Youngblood, M. Karpov, H. Gehring, X. Li, M. Stappers, M. Le Gallo, X. Fu, A. Lukashchuk, A. S. Raja, J. Liu, C. D. Wright, A. Sebastian, T. J. Kippenberg, W. H. P. Pernice, H. Bhaskaran, Parallel convolutional processing using an integrated photonic tensor core. *Nature* **589**, 52–58 (2021).

10. M. Miscuglio, V. J. Sorger, Photonic tensor cores for machine learning. *Appl. Phys. Rev.* **7**, 031404 (2020).

11. B. Dong, S. Aggarwal, W. Zhou, U. E. Ali, N. Farmakidis, J. S. Lee, Y. He, X. Li, D.-L. Kwong, C. D. Wright, W. H. P. Pernice, H. Bhaskaran, Higher-dimensional processing using a photonic tensor core with continuous-time data. *Nat. Photon.* **17**, 1080–1088 (2023).

12. J. Cheng, Y. Xie, Y. Liu, J. Song, X. Liu, Z. He, W. Zhang, X. Han, H. Zhou, K. Zhou, H. Zhou, J. Dong, X. Zhang, Human emotion recognition with a microcomb-enabled integrated optical neural network. *Nanophotonics* **12**, 3883–3894 (2023).

13. Y. Huang, T. Fu, H. Huang, S. Yang, H. Chen, Sophisticated deep learning with on-chip optical diffractive tensor processing. *Photon. Res.* **11**, 1125–1138 (2023).

14. M. Nakajima, K. Tanaka, T. Hashimoto, Scalable reservoir computing on coherent linear photonic processor. *Commun. Phys.* **4**, 20 (2021).

15. T. Hülser, F. Köster, L. Jaurigue, K. Lüdge, Role of delay-times in delay-based photonic reservoir computing [Invited]. *Opt. Mater. Express* **12**, 1214–1231 (2022).

16. J. Zha, S. Shi, A. Chaturvedi, H. Huang, P. Yang, Y. Yao, S. Li, Y. Xia, Z. Zhang, W. Wang, H. Wang, S. Wang, Z. Yuan, Z. Yang, Q. He, H. Tai, E. H. T. Teo, H. Yu, J. C. Ho, Z. Wang, H. Zhang, C. Tan, Electronic/optoelectronic memory device enabled by tellurium-based 2D van der Waals heterostructure for in-sensor reservoir computing at the optical communication band. *Adv. Mater.* **35**, 2211598 (2023).

17. Y. Eliezer, U. Rührmair, N. Wisiol, S. Bittner, H. Cao, Tunable nonlinear optical mapping in a multiple-scattering cavity. *Proc. Natl. Acad. Sci. U.S.A.* **120**, e2305027120 (2023).

18. S. Becker, D. Englund, B. Stiller, An optoacoustic field-programmable perceptron for recurrent neural networks. *Nat. Commun.* **15**, 3020 (2024).

19. A. Hazan, B. Ratzker, D. Zhang, A. Katiyi, M. Sokol, Y. Gogotsi, A. Karabchevsky, MXene-nanoflakes-enabled all-optical nonlinear activation function for on-chip photonic deep neural networks. *Adv. Mater.* **35**, 2210216 (2023).

20. K. Bi, L. Xie, H. Zhang, X. Chen, X. Gu, Q. Tian, Accurate medium-range global weather forecasting with 3D neural networks. *Nature* **619**, 533–538 (2023).

21. H. H. Zhu, J. Zou, H. Zhang, Y. Z. Shi, S. B. Luo, N. Wang, H. Cai, L. X. Wan, B. Wang, X. D. Jiang, J. Thompson, X. S. Luo, X. H. Zhou, L. M. Xiao, W. Huang, L. Patrick, M. Gu, L.

C. Kwek, A. Q. Liu, Space-efficient optical computing with an integrated chip diffractive neural network. *Nat. Commun.* **13**, 1044 (2022).

22. F. Ashtiani, A. J. Geers, F. Aflatouni, An on-chip photonic deep neural network for image classification. *Nature* **606**, 501–506 (2022).

23. X. Xu, M. Tan, B. Corcoran, J. Wu, A. Boes, T. G. Nguyen, S. T. Chu, B. E. Little, D. G. Hicks, R. Morandotti, A. Mitchell, D. J. Moss, 11 TOPS photonic convolutional accelerator for optical neural networks. *Nature* **589**, 44–51 (2021).

24. B. J. Shastri, A. N. Tait, T. Ferreira de Lima, W. H. P. Pernice, H. Bhaskaran, C. D. Wright, P. R. Prucnal, Photonics for artificial intelligence and neuromorphic computing. *Nat. Photon.* **15**, 102–114 (2021).

25. Z. Xu, T. Zhou, M. Ma, C. Deng, Q. Dai, L. Fang, Large-scale photonic chiplet Taichi empowers 160-TOPS/W artificial general intelligence. *Science* **384**, 202–209 (2024).

26. A. Song, S. N. Murty Kottapalli, R. Goyal, B. Schölkopf, P. Fischer, Low-power scalable multilayer optoelectronic neural networks enabled with incoherent light. *Nat. Commun.* **15**, 10692 (2024).

27. F.-C. F. Tsai, C. J. O'Brien, N. S. Petrović, A. D. Rakić, Analysis of optical channel cross talk for free-space optical interconnects in the presence of higher-order transverse modes. *Appl. Optics* **44**, 6380–6387 (2005).

28. T. Fu, J. Zhang, R. Sun, Y. Huang, W. Xu, S. Yang, Z. Zhu, H. Chen, Optical neural networks: Progress and challenges. *Light Sci. Appl.* **13**, 263 (2024).

29. Y. Shen, N. C. Harris, S. Skirlo, M. Prabhu, T. Baehr-Jones, M. Hochberg, X. Sun, S. Zhao, H. Larochelle, D. Englund, M. Soljačić, Deep learning with coherent nanophotonic circuits. *Nat. Photon.* **11**, 441–446 (2017).

30. M. A. Nahmias, T. F. d. Lima, A. N. Tait, H. T. Peng, B. J. Shastri, P. R. Prucnal, Photonic multiply-accumulate operations for neural networks. *IEEE J. Sel. Top. Quantum Electron.* **26**, 1–18 (2020).

31. A. N. Tait, T. Ferreira de Lima, M. A. Nahmias, H. B. Miller, H.-T. Peng, B. J. Shastri, P. R. Prucnal, Silicon photonic modulator neuron. *Phys. Rev. Appl.* **11**, 064043 (2019).

32. A. de Vries, The growing energy footprint of artificial intelligence. *Joule* **7**, 2191–2194 (2023).

33. R. Landauer, Irreversibility and heat generation in the computing process. *IBM J. Res. Dev.* **5**, 183–191 (1961).

34. E. Masanet, A. Shehabi, N. Lei, S. Smith, J. Koomey, Recalibrating global data center energy-use estimates. *Science* **367**, 984–986 (2020).

35. V. Y. Kreinovich, Arbitrary nonlinearity is sufficient to represent all functions by neural networks: A theorem. *Neural Netw.* **4**, 381–383 (1991).

36. K. Hornik, Approximation capabilities of multilayer feedforward networks. *Neural Netw.* **4**, 251–257 (1991).

37. K. Hornik, M. Stinchcombe, H. White, Multilayer feedforward networks are universal approximators. *Neural Netw.* **2**, 359–366 (1989).

38. G. Cybenko, Approximation by superpositions of a sigmoidal function. *Math. Control Signal System* **2**, 303–314 (1989).

39. P. Weinberger, John Kerr and his effects found in 1877 and 1878. *Philos. Mag. Lett.* **88**, 897–907 (2008).

40. T. Yan, J. Wu, T. Zhou, H. Xie, F. Xu, J. Fan, L. Fang, X. Lin, Q. Dai, Fourier-space diffractive deep neural network. *Phys. Rev. Lett.* **123**, 023901 (2019).

41. M. Yildirim, I. Oguz, F. Kaufmann, M. R. Escalé, R. Grange, D. Psaltis, C. Moser, Nonlinear optical feature generator for machine learning. *APL Photonics* **8**, 106104 (2023).

42. Q. Glorieux, T. Aladjidi, P. D. Lett, R. Kaiser, Hot atomic vapors for nonlinear and quantum optics. *N. J. Phys.* **25**, 051201 (2023).

43. M. Miscuglio, Z. Hu, S. Li, J. K. George, R. Capanna, H. Dalir, P. M. Bardet, P. Gupta, V. J. Sorger, Massively parallel amplitude-only Fourier neural network. *Optica* **7**, 1812 (2020).

44. R. Hamerly, L. Bernstein, A. Sludds, M. Soljačić, D. Englund, Large-scale optical neural networks based on photoelectric multiplication. *Phys. Rev. X* **9**, 021032 (2019).

45. Y. Zuo, B. Li, Y. Zhao, Y. Jiang, Y.-C. Chen, P. Chen, G.-B. Jo, J. Liu, S. Du, All-optical neural network with nonlinear activation functions. *Optica* **6**, 1132–1137 (2019).

46. M. Sheik-Bahae, A. A. Said, T. H. Wei, D. J. Hagan, E. W. V. Stryland, Sensitive measurement of optical nonlinearities using a single beam. *IEEE J. Quantum Electron.* **26**, 760–769 (1990).

47. F. Xia, K. Kim, Y. Eliezer, S. Han, L. Shaughnessy, S. Gigan, H. Cao, Nonlinear optical encoding enabled by recurrent linear scattering. *Nat. Photon.* **18**, 1067–1075 (2024).

48. M. Yildirim, N. U. Dinc, I. Oguz, D. Psaltis, C. Moser, Nonlinear processing with linear optics. *Nat. Photon.* **18**, 1076–1082 (2024).

49. I.-C. Khoo, *Liquid Crystals* (John Wiley & Sons, 2022).

50. Y. LeCun, L. Bottou, Y. Bengio, P. Haffner, Gradient-based learning applied to document recognition. *Proc. IEEE* **86**, 2278–2324 (1998).

51. R. O'Donnell, *Analysis of Boolean Functions* (Cambridge Univ. Press, 2014).

52. R. Kohavi, paper presented at the 14th International Joint Conference on Artificial Intelligence, Montreal, Canada, 20 August 1995.

53. M. W. Browne, Cross-validation methods. *J. Math. Psychol.* **44**, 108–132 (2000).

54. F. Rosenblatt, The perceptron: A probabilistic model for information storage and organization in the brain. *Psychol. Rev.* **65**, 386–408 (1958).

55. M. Marvin, A. P. Seymour, B. Leon, “Perceptrons,” in *Perceptrons: An Introduction to Computational Geometry* (MIT Press, 2017), pp. i–xxiii.

56. D. E. Rumelhart, G. E. Hinton, R. J. Williams, Learning representations by back-propagating errors. *Nature* **323**, 533–536 (1986).

57. X. Lin, Y. Rivenson, N. T. Yardimci, M. Veli, Y. Luo, M. Jarrahi, A. Ozcan, All-optical machine learning using diffractive deep neural networks. *Science* **361**, 1004–1008 (2018).

58. C. Qian, X. Lin, X. Lin, J. Xu, Y. Sun, E. Li, B. Zhang, H. Chen, Performing optical logic operations by a diffractive neural network. *Light Sci. Appl.* **9**, 59 (2020).

59. O. A. Marino, A. Juanicotena, J. Errasti, D. Mayoral, F. Manrique de Lara, R. Vinuesa, E. Ferrer, A comparison of neural-network architectures to accelerate high-order h/p solvers. *Phys. Fluids* **36**, 107132 (2024).

60. C. L. Giles, T. Maxwell, Learning, invariance, and generalization in high-order neural networks. *Appl. Optics* **26**, 4972–4978 (1987).

61. L. Deng, The MNIST database of handwritten digit images for machine learning research [best of the web]. *IEEE Signal Process. Mag.* **29**, 141–142 (2012).

62. G. Cohen, S. Afshar, J. Tapson, A. Van Schaik, EMNIST: Extending MNIST to handwritten letters, in *2017 International Joint Conference on Neural Networks (IJCNN)* (IEEE, 2017), pp. 2921–2926.

63. K. Nazarpour, M. Chen, *Handwritten Chinese Numbers* (Newcastle Univ. Data, 2017).

64. L. Mandel, E. Wolf, *Optical Coherence and Quantum Optics* (Cambridge Univ. Press, 1995).

65. I. A. D. Williamson, T. W. Hughes, M. Minkov, B. Bartlett, S. Pai, S. Fan, Reprogrammable electro-optic nonlinear activation functions for optical neural networks. *IEEE J. Sel. Top. Quantum Electron.* **26**, 1–12 (2020).

66. J. R. Basani, M. Heuck, D. R. Englund, S. Krastanov, All-photonic artificial-neural-network processor via nonlinear optics. *Phys. Rev. Appl.* **22**, 014009 (2024).

67. C. Chen, Z. Yang, T. Wang, Y. Wang, K. Gao, J. Wu, J. Wang, J. Qiu, D. Tan, Ultra-broadband all-optical nonlinear activation function enabled by MoTe2/optical waveguide integrated devices. *Nat. Commun.* **15**, 9047 (2024).

68. A. Dimitriou, M. Hu, J. Hare, G. V. Merrett, paper presented at the 2023 Design, Automation & Test in Europe Conference & Exhibition (DATE), 17–19 April 2023.

69. P. SK, S. A. Kesanapalli, Y. Simmhan, Characterizing the performance of accelerated jetson edge devices for training deep learning models. *Proc. ACM Meas. Anal. Comput. Syst.* **6**, 1–26 (2022).

70. H. Mahmud, P. Kang, K. Desai, P. Lama, S. K. Prasad, paper presented at the 2024 IEEE International Parallel and Distributed Processing Symposium Workshops (IPDPSW), 27–31 May 2024.

71. C. Liu, Q. Ma, Z. J. Luo, Q. R. Hong, Q. Xiao, H. C. Zhang, L. Miao, W. M. Yu, Q. Cheng, L. Li, T. J. Cui, A programmable diffractive deep neural network based on a digital-coding metasurface array. *Nat. Electron.* **5**, 113–122 (2022).

72. K. Matsushima, T. Shimobaba, Band-limited angular spectrum method for numerical simulation of free-space propagation in far and near fields. *Opt. Express* **17**, 19662–19673 (2009).
